# Editorial: Women in veterinary neurology and neurosurgery: 2021

**DOI:** 10.3389/fvets.2023.1147908

**Published:** 2023-02-10

**Authors:** Luisa De Risio

**Affiliations:** Linnaeus Veterinary Limited, Shirley, United Kingdom

**Keywords:** dog, cat, neurology—clinical, neurosurgical procedures, editorial

This Frontiers in Veterinary Science Women in Veterinary Neurology and Neurosurgery collection of scientific articles presents the innovative work of female neurologists working in the UK, Europe, and North America. The series comprises 13 articles showcasing original research and case reports in various fields of Veterinary Neurology and Neurosurgery, including advances in diagnostic imaging techniques and novel neurosurgical procedures.

Diffusion MRI is a specific sequence that detects and quantifies water diffusivity, which is the molecular motion (Brownian movement) of water and represents an intrinsic feature of tissues. Diffusion-weighted imaging (DWI) and apparent diffusion coefficient (ADC) are MRI sequences routinely used in the diagnostic investigation of suspected cerebrovascular accidents (CVA) in people (1). There is limited information on diffusion-weighted characteristics of naturally occurring CVA in dogs. Extrapolation from other species has limitations due to differences in how CVA appears on DWI over time (i.e., from the onset of CVA to the time of MRI) (2). The retrospective study by Boudreau et al. describes DWI MRI findings of spontaneous canine CVA in relation to the time of clinical onset, the DWI type (EPI vs. non-EPI), and the presence or absence of a haemorrhagic component of the lesion. The results of this study help to inform the appropriate clinical interpretation of these sequences by veterinary neurologists and neuroradiologists.

Steroid-responsive meningitis-arteritis (SRMA) is an inflammatory disorder of probable immune mediated origin, commonly recognized in dogs (3). It typically affects young, medium to large breed dogs and can present as an acute or chronic condition (3). The acute form of SRMA is characterized by cervical hyperalgesia, pyrexia, and cerebrospinal fluid (CSF) neutrophilic pleocytosis (4). Some biomarkers have been used to understand the pathogenesis of SRMA and support its diagnosis (5, 6). Clinical and laboratory findings in dogs with a chronic form of SRMA are less specific than those observed in the acute form, making the diagnosis more challenging. The MRI finding of SRMA in dogs include enhancement of the meninges in the cervical region on T1-weighted (T1W) images after intravenous injection of gadolinium, and signal changes of the cervical muscle such as hyperintensity on T2-weighted (T2W) and short tau inversion recovery (STIR) (7), as well as contrast enhancement. The study by Remelli et al. highlights the usefulness of low and high-field MRI in complementing clinical and laboratory findings in the diagnosis of SRMA. In this retrospective study, including 70 dogs with SRMA, the MRI showed abnormalities in 98.6% of dogs, with the majority (87.1%) being MRI features suggestive of meningeal inflammation. T1W FAT-SAT sequences were particularly useful in detecting meningeal enhancement. In addition, the contrast enhancement of the synovium of the cervical articular facets and the epaxial muscles was detected in 48.6% of dogs.

Spinal epidural empyema (SEE) is characterized by the “accumulation of purulent material in the epidural space of the vertebral canal” (8). It can result in neurological deficits and even death if untreated or unresponsive to treatment (9). There is limited information on canine SEE in veterinary literature. A study by Laws et al. describes the clinical and diagnostic findings, treatment (conservative or surgical), and outcomes of dogs with SEE that were presented to five referral hospitals in the UK. This study provides detailed information on the presenting clinical signs, MRI findings, laboratory investigation results, treatment, and long-term outcomes. The results of this study inform client communication and clinical decisions on canine SSE management.

Various neurological conditions such as vertebral fractures and luxations, malformations, neoplasia, and intervertebral disc degenerative and infectious disorders can result in instability of the vertebral column. Different surgical techniques have been described to stabilize the vertebral column of dogs and cats. These procedures require specialistic equipment combined with advanced neurosurgical skills and expertise. One of the main risks of spinal stabilization is the violation of neurovascular structures during implant placement resulting in serious and potentially irreversible neurologic deficits for the patient. Reliable intra and post-operative evaluation of the accuracy of implant placement is necessary for the safe and successful treatment of patients undergoing stabilization of the vertebral column (10). A canine cadaveric imaging and anatomic study by Goffart et al. shows that end-on fluoroscopy, with or without inversion, is a highly accurate technique for the intraoperative evaluation of bicortically placed Steinmann pins' position in the canine thoracolumbar vertebral column. Three-dimensionally printed patient-specific drill guides have been used to improve the accuracy of implant placement in the canine spine (11).

Three-dimensionally printed patient-specific drill guides have recently been proposed to assist with another challenging neurosurgical procedure that involves a steep learning curve. The study by Escauriaza et al. investigated the accuracy of a 3-dimensional dog-specific printed surgical guide to support the surgeon performing transsphenoidal hypophysectomy, which is performed as surgical treatment of pituitary-dependent hyperadrenocorticism. Pituitary-dependent hyperadrenocorticism is a chronic and progressive disorder (12) that can be treated medically or surgically with or without radiation therapy. Surgical treatment to remove the pituitary neoplasm is increasingly used in dogs. However, this surgical procedure is challenging and requires prolonged training and advanced expertise. The methods described in the study by Escauriaza et al. support the identification of the area to drill in the basisphenoid bone to access the salle turcica and the pituitary mass.

This collection of articles promotes the work of female neurology and neurosurgery researchers and contributes to the advancement of diagnosis and treatment of different neurologic conditions (i.e., infectious, inflammatory, neoplastic) of the central and peripheral nervous system.

## Author contributions

The author confirms being the sole contributor of this work and has approved it for publication.
